# Evaluating the impact of an established Telecare system on secondary care usage

**Published:** 2012-06-15

**Authors:** Will Sopwith

**Affiliations:** NHS Wirral, UK

**Keywords:** patients at risk of re-hospitalisation, PARR, admissions data, length of stay, integration

## Abstract

**Objectives:**

The Wirral Assistive Technology Programme (WAT) has operated across the whole population of the Borough for 3 years. It includes the installation of telecare technologies such as falls alerts and movement sensors to prolong users’ independence and reduce use of secondary care. Qualitative evaluation of user and carer perspectives indicated a reduction in admissions as a result of the technology and we sought to demonstrate this at a population level.

**Methods:**

Secondary care episodes for A&E, admissions and outpatients were temporally analysed to identify changed trend in secondary care usage as a result of installation for 400 users. Primary diagnoses at point of secondary care engagement were categorised to discern those that might have been mediated by WAT (e.g. falls and fractures) from those likely to be incidental (e.g. infection). The nature of the WAT service made the identification of a control group challenging and it was necessary to try and establish the counterfactual of secondary care usage in an equivalent group of patients not engaged with WAT. We used Patients at Risk of Re-Hospitalisation (PARR) scores which are routinely assigned to every patient discharge in Wirral to identify an equivalent group of patients not engaged with WAT and compared trends of secondary care usage as above. Controls were selected on the basis of an equivalent PARR score to an AT user on the same date as that user’s final discharge before installation of AT.

**Results:**

A&E and inpatient episodes showed regression to the mean around the point of AT installation, both for AT-related and unrelated diagnoses (between 660 days pre-installation and 240 days post-). Outpatient episodes showed a stable trend. We found no evidence that AT installation reduced the average number of secondary care episodes per patient, reduced the average PARR score or reduced the average length of hospital stay in comparison to controls.

**Conclusions:**

Although conceptually and qualitatively WAT should be impacting secondary care episodes, we could find no evidence of this. This has important implications for the way the programme is currently integrated with other local re-ablement schemes, how it is monitored and what incentives there are in the discharge system to promote its use. This paper will outline actions taken to embed the programme since the evaluation was completed (September 2011).

